# The Chick Embryo as an Experimental System for Melanoma Cell Invasion

**DOI:** 10.1371/journal.pone.0053970

**Published:** 2013-01-14

**Authors:** Christian Busch, Jelena Krochmann, Ulrich Drews

**Affiliations:** Section of Dermato-Oncology, Department of Dermatology, University of Tuebingen, Tuebingen, Germany; University Hospital Hamburg-Eppendorf, Germany

## Abstract

**Background:**

A primary cutaneous melanoma will not kill the patient, but its metastases. Since in vitro studies on melanoma cells in 2-D cultures do often not reflect reality, 3-D models might come closer to the physiological situation in the patient during cancer initiation and progression.

**Methodology/Principal Findings:**

Here, we describe the chick embryo model for in vivo studies of melanoma cell migration and invasion. After transplantation of neural crest-derived melanoma cells into the neural tube, the melanoma cells resume neural crest cell migration along the medial and lateral pathways and finally undergo apoptosis in the target areas. Upon transplantation into ectopic areas such as the hindbrain or the optic cup malignant invasion and local tissue destruction occurs. In contrast, melanocytes are not able to spontaneously resume neural crest cell migration. However, malignant invasion can be induced in melanocytes by pre-treatment with the TGF-beta family members bone morphegenetic protein-2 or nodal. Transplantation of MCF7 breast cancer cells yields a different growth pattern in the rhombencephalon than melanoma cells.

**Conclusions/Significance:**

The chick embryo model is a feasible, cost-effective in vivo system to study invasion by cancer cells in an embryonic environment. It may be useful to study invasive behavior induced by embryonic oncogenes and for targeted manipulation of melanoma or breast cancer cells aiming at ablation of invasive properties.

## Introduction

Cutaneous melanoma is a highly aggressive malignancy with increasing incidence, limited therapeutic options in the metastatic stage of disease and a reduced overall survival of 6–9 months in untreated patients and to 5 months after occurrence of brain metastases [1], [2].

Considering the crucial importance of cellular migration (leading to metastasis) for patient survival, it seems odd that in the past decades, therapeutic approaches for stage IV metastatic disease mainly focused on interference with melanoma cell proliferation (chemotherapy, radiation), on immunological stimulation (vaccination, blocking of CTLA-4), or on oncogene-targeted therapy (e.g. BRAF V600E mutation [3]) available only for a subpopulation of melanomas. Melanoma cells can perform a “phenotype switching” from a proliferating to a migrating state and vice versa [4]. The current lack of drugs specifically inhibiting melanoma cell migration is in part due to the lack of suitable in vivo models able to mimic the complex 3D-in vivo situation that melanoma cells have to cope with in the patient. The initiation process of cellular invasion in melanoma might be a common feature in all melanomas via up-regulation of early embryonic genes such as Notch1 [5] and nodal [6], or via up-regulation of neural crest signaling [7].

Various genetically modified mouse models are used in melanoma research to study melanomas generation and progression (e.g. Hgf-Cdk4(R24C) mice [8]) or as a model for subcutaneous tumor nodule formation [9]. Although of eminent importance for the testing of novel drugs targeting pathways involved in melanoma cell proliferation or to induce an immune reaction directed against such experimentally generated melanomas, the mouse models seem limited to this application range.

The chick embryo as experimental system has several advantages. The embryo in the egg is easily accessible. Transplants are not rejected, because the immune system has not yet developed. Legal and ethical restrictions are limited to the stages before and after hatching. Classical grafting onto the chorioallantoic membrane (CAM) at embryonic day 10 (E10) was used to study primary melanoma growth and metastasis [10]. Chambers et al., [11] injected B16F1 melanoma cells into both the veins of the chorioallantoic membrane of E11 chick embryos and the tail vein of mice and examined tumor formation after seven days in chick embryos and after 20 days in mice. The number of tumors for a given number of cells injected was higher in the chick than in the mouse. B16F1 tumors grew in most embryonic chick organs while their growth in the mouse was restricted primarily to the lungs. The chick embryo was also used as model for uveal melanoma [12]. Human uveal or skin melanoma cells were injected into the optic cup at day E3.5 and tumor growth was followed up to E19.

In our experimental system we use the early chick embryo in the primitive streak and somite stages (E2–E5) and transplant the melanoma cells into their site of origin, the neural crest, or into ectopic sites, the optic cup or the brain vesicles. Malignant growth can be interpreted as untimely and ectopic re-activation of embryonic genes in adult quiescent stem cell populations. Embryonic genes, transcription factors, and transduction chains regulate cell migration and proliferation in the embryo and become inactivated during differentiation. Re-activation in the adult is associated with malignant growth. Our approach is to bring the melanoma cells back into the original embryonic environment, where the re-activated oncogenes may fulfill their original tasks. Our results indicate, that after transplantation of melanoma cells into their autochthonous environment, the neural crest, the oncogenes can be tamed, and the melanoma cells undergo apoptosis, whereas in ectopic sites they exhibit malignant growth.

In 1998, we presented for the first time the embryonic neural tube as site for melanoma cell transplantation [13]. We transplanted SKMel28 melanoma cells into the lumen of the neural tube and observed a spontaneous integration into the neural crest with subsequent physiological neural crest cell migration of the transplanted melanoma cells [14]. Neural crest cell migration becomes directly visible in the live embryo, when the GFP labeled B16-F1 mouse melanoma cell line is used [15]. The capability to resume neural crest cell migration depends on the constitutive production of BMP-2 (bone morphogenetic protein-2) and can be ablated by pre-treatment of melanoma cells with the embryonic BMP antagonist noggin [16]. After transplantation into the optic cup the melanoma cells exhibit malignant invasive growth, which also is ablated by pre-treatment with noggin [17].

Here, the technical aspects of the chick embryo model are presented in detail including step-by-step instructions and pitfalls. The capabilities are exemplified by a brief summary of our original experiments supplemented by new data on transplantation of non-transformed primary human melanocytes into the neural crest and into the optic cup, and on malignant invasive growth of melanoma and breast cancer cells in the hindbrain as novel model for invasive brain metastasis.

## Materials and Methods

### Ethics Statement

According to German animal care guidelines, no IACUC approval was necessary to perform the embryo experiments. According to the local guidelines, only experiments with chick embryos E18 and older need IACUC approval. However, the embryos used in this study were all in early stages of embryonic development (between E2 and E7).

### Preparation of Eggs and Transplantation of Cells

**Figure 1:**
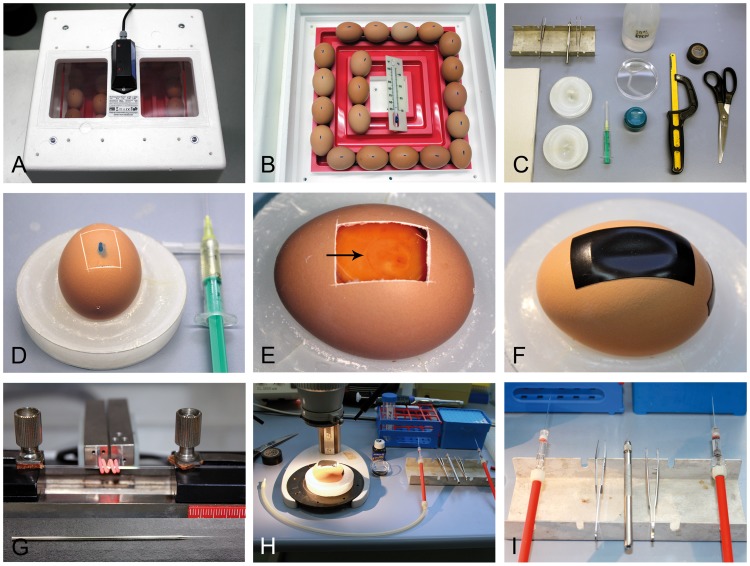
Egg preparation and fenestration. (A) Incubator containing eggs. (B) The top of each egg is marked to indicate the location of the embryo (blastoderm). (C) Tools required for fenestration of the eggs are depicted: (from left to right) paper towels, tungsten needle and forceps, 2 egg holding devices, syringe with needle, 80% ethanol, plastic petri dish for egg shell waste, egg piercer, hacksaw, adhesive tape, scissors. (D) A pre-determined breaking point is generated after removal of 2 ml albumen to lower the level of the blastoderm. (E) After removal of the shell, the embryo is discernible on the blastoderm (arrow). (F) The egg is sealed with adhesive tape and re-incubated. (G) Capillaries are pulled before transplantation. (H) Working place (stereo-microscope with epi-illumination, diluted black ink, PBS, pipette) and (I) tools (mouth pipette, forceps, tungsten needle) required for transplantation of the cells.

Fertilized eggs of leghorn chickens (Gallus gallus domesticus) were obtained from a hatchery (Weiss, Kilchberg, Germany) and incubated at 38°C in a temperature-controlled brooder (BRUJA Type 400a, Brutmaschinen Janeschitz, Hammelburg, Germany) (Figure 1A) without rolling. The uppermost spot of the eggshell (and thus indirectly the blastoderm, which is always oriented towards the top part of the egg) was marked on each egg with a permanent marker (Figure 1B).

**Figure 2:**
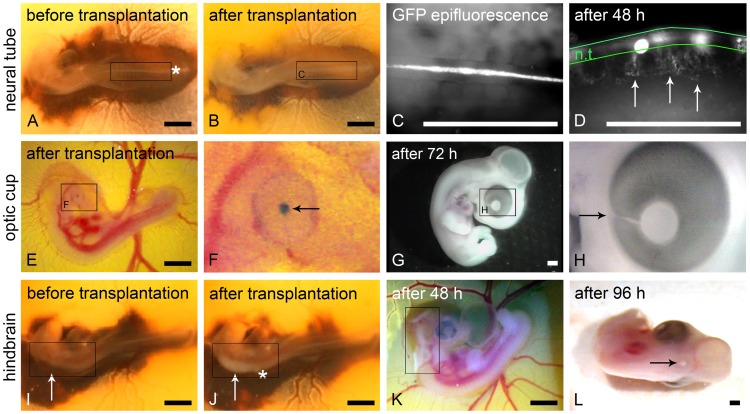
Transplantation of melanoma cells into three distinct niches of the chick embryo. (A) Chick embryo stage 12–13 HH before and (B) directly after transplantation of B16-F1 melanoma cells into the neural tube. The entering site of the micro-pipette is marked (asterisk in A). Note the dilated neural tube (frame in B) due to the transplanted cells when compared to (A). (C) B16-F1 cells can be detected via GFP epifluorescence in the lumen of the neural tube directly after transplantation. (D) 48 h after transplantation ventrally emigrating B16-F1 cells are clearly discernible (arrows) in lateral view; the borders of the neural tube are outlined in green. (E) Chick embryo stage 19 HH directly after transplantation of B16-F1 melanoma cell aggregates into the optic cup. (F) Aggregates were stained with nile blue sulphate before transplantation for better visibility. Higher magnification shows the aggregates behind the embryonic lens (arrow). A temporary capillary bleeding can be discerned at the injection spot at the choroid fissure in (F). (G) Macroscopically no tumor growth is visible 72 h after transplantation. (H) The former entering site of the micro-pipette (choroid fissure) is marked (arrow). (I) Chick embryo stage 12–13 HH before and (J) directly after transplantation of human melanoma cells into the ventricle of the hindbrain (rhombencephalon, frame in I). The entering site of the micro-pipette is marked with the asterisk in (J). Note the melanoma cell-filled brain ventricle (frame in J). (K) 48 h after transplantation a growing tumor is already visible in the hindbrain (frame). (L) After 96 h a single condensed tumor is visible in the dorsal midline of the neural epithelium (arrow). Scale bars in A, B and E–L: 1 mm; scale bars in C and D: 0.5 mm.

For transplantation of melanoma cells into the neural tube, eggs were prepared after 48 h of incubation (stage 12–13 according to Hamburger and Hamilton (HH) [18]). The equipment necessary for fenestration is shown in Figure 1C. First, the eggs were sprayed with 80% ethanol to reduce surface contamination. They were then placed into previously prepared holding devices (Figure 1D) consisting of a plastic Petri dishes filled with paraffin containing a cast of the egg. Next, a small hole was pierced into the lateral edge of the egg using a classic egg piercer (the blue object next to the hacksaw in Figure 1C) and 2 ml of albumen were withdrawn with a syringe (Injekt ® 2 ml, B. Braun Melsungen AG, Germany; needle used: BD Microlance 3, 20G×1½ inch, Becton, Dickinson and Company, Franklin Lakes, NJ, USA) to lower the level of the blastoderm. The egg was then prepared for fenestration by using a high speed steel blade hacksaw (250 mm, 15–802; Stanley, New Britain, Australia) to generate a rectangular predetermined breaking point on the shell around the previously marked spot (about 15×25 mm in size) (Figure 1D). Next, the “window” was opened by removal of the eggshell with bent forceps. The embryo is in the somite stage and visible on top of the yolk (Figure 1E). The egg was then sealed with adhesive tape (Super88, 3 M, St. Paul, MN) and replaced into the incubator (Figure 1F). For transplantation, freshly pulled capillaries from Kwik-Fil™ Borosilicate Glass (World Precision Instruments, Inc., Sarasota, FL) were prepared with a capillary puller (H. Saur Laborbedarf, Reutlingen, Germany), as shown in Figure 1G. The working environment under the stereomicroscope (Zeiss, Oberkochen, Germany) with epi-illumination (Schott, Mainz, Germany), the mouth pipettes and required instruments on a sterile bench are depicted in Figures 1H and I.

For better visualization Black Ink A diluted in PBS (Pelikan, Hannover, Germany) was injected with a glass pipette between yolk and embryo (Figures 2A and 2I). For each series of transplantation, one of the following cells were used as aggregates or cell suspensions: Mouse B16-F1 metastatic melanoma cells (gifted from [19]); human SKMel28 metastatic melanoma cells (purchased as part of the NCI60 panel of cancer cells from the NCI); human 451LU metastatic melanoma cells (gifted from Meenhard Herlyn, Wistar Institute, Philadelphia, USA [20]), or human melanocytes (human epidermal melanocytes neonatal (HEMn), CellSystems, Troisdorf, Germany, cultivated in Lifeline's DermaLife M medium (CellSystems)). The melanoma cells were cultivated as described previously [16]. MCF7 breast cancer cells (purchased as part of the NCI60 panel of cancer cells from the NCI) were cultivated in the same conditions as the melanoma cells [16]. Cells were injected into the lumen of the neural tube by entering caudally at the site of the tail bud to prevent tissue damage. Injections were performed at stages 12–13 HH, during or shortly after closure of the neural tube (Figure 2B). In the case of GFP-labeled B16-F1 cells, GFP epifluorescence was used to demonstrate the site-specific transplantation result (Figure 2C). Embryos were further incubated for 48h; GFP epifluorescence illustrated dorso-ventrally migrating melanoma cells in lateral view of the embryo (Figure 2D).

At stage 20 HH the embryonic optic cup is localized at the surface of the chorioallantoic membrane and easily recognized because the pigment epithelium has just developed. For transplantation into the optic cup, eggs were fenestrated after 72–80 h of incubation (corresponding to stage 19–20 HH). B16-F1 aggregates or melanocyte aggregates (untreated, bone morphogenetic protein (BMP)-2 pre-treated or nodal pre-treated; n = 7 embryos per group) were transplanted into the optic cup (Figures 2E, F and Table 1), entering at the site of the choroid fissure of the optic cup (pointed out in Figure 2H). In some cases, local capillary bleeding occurred, which usually stopped within 1–2 min without disrupting embryo development. For better visibility and documentation purposes, B16-F1 melanoma cell aggregates were stained with nile blue sulphate before transplantation (Bayer, Leverkusen, Germany). After transplantation, the aggregates remained at the site of transplantation and were documented. Eggs were sealed with adhesive tape and further incubated for 72 h (Figure 2G).

**Table 1:** Evaluation of melanocyte invasion in the optic cup.

Treatment	Embryo	Injectionchannel	Choroid	Hyaloidvessels	Vitreousbody	Behindlens/lens	Otherinvasive
Untreated	1	x		x		x	
	2	x			x	x	
	3			x	x	x	
	4			x	x		
	5				x	x	
	6			x	x		
	7	x		x	x	x	
BMP-2	1		x (invasive)	x	x		retina
	2				x	x	
	3			x	x		
	4		x (invasive)		x	invasive	
	5			x (invasive)	x	x	
	6				x	x	
	7			x (invasive)			
Nodal	1					x	
	2					x	
	3			x	x	x	
	4					x	
	5				x	x	
	6			x		x	
	7		x (invasive)	x (invasive)	x		

For evaluation of invasive migration, the melanocytes (identified by their specific pigmentation) were filed according to the embryonic micro-compartments in which they were found in the histological serial sections: injection channel, choroid, hyaloid vessels, vitreous body, and behind the lens. “Invasive” refers to single melanocytes found in locations other than the spot of injection, invading the host tissues. “Other invasive” refers to single invasive melanocytes that were found in micro-compartments other than the listed ones.

For transplantation into the brain ventricles, the capillary was entered into the embryo cranially at the most caudal site of the rhombencephalon (Figures 2I, J), and embryos were incubated for additional 48 or 96 h (Figures 2 K, L). 95% of the embryos that were transplanted into the neural tube, and 80% of the embryos that were transplanted into the brain ventricles or into the optic cup survived the transplantation procedure and the following re-incubation time ranging between 24 and 96 h.

### Fixation of Embryos and Paraffin Embedding

At the end of the incubation period, embryos were removed from the eggs using forceps and bent scissors (Moria, Antony, France). Embryos that had received transplantations into the optic cup were decapitated. Entire embryos and embryo heads were fixed in 4% buffered paraformaldehyde for 12–24 h depending on the size of the embryo and were transferred into tissue cassettes (Rotilabo® Macro, Carl Roth, Karlsruhe, Germany). After rinsing with water, samples were dehydrated with ethanol, treated with xylene and embedded in paraplast in a routine histology embedding automat. The final casting in the paraffin block is crucial for future histological evaluation and therefore was performed in a similar manner in all embryos. It was determined, whether transverse or longitudinal serial sections of the site of transplantation yielded the best results. In the presented cases, transverse sections were chosen in most cases to get a full overview of the embryo permitting a depiction of both the medial and lateral neural crest cell pathways.

### Species Specific Markers

**Figure 3:**
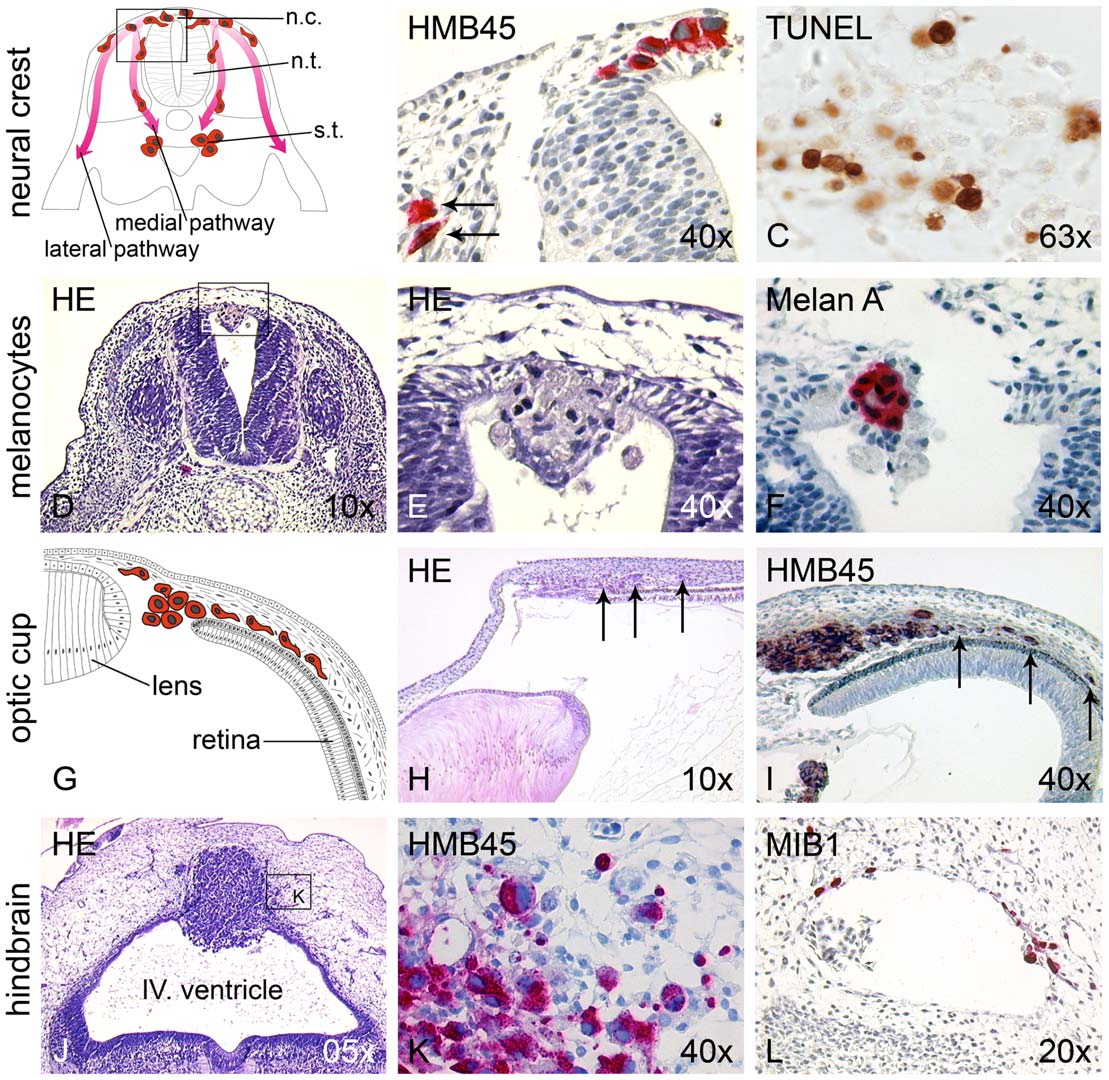
Histology, immunohistochemistry and in situ hybridization of the chick embryos. (A) Schematic drawing depicting ventral and medial neural crest migration pathways. n.c. neural crest; n.t. neural tube; s.t. sympathetic trunk. (B) Chick embryo 24 h after transplantation of SKMel28 melanoma cells into the neural tube. Melanoma cells (visualized by HMB45 immunoreactivity) spontaneously resuming neural crest migration have a stretched, mesenchymal-like morphology (arrows). (C) At the site of destination along the ventral migration pathway (para-aortic sympathetic ganglia) melanoma cells undergo apoptosis, visualized by TUNEL staining. (D,E) Chick embryo 24 h after transplantation of benign primary human melanocytes into the neural tube. Melanocytes (showing a compact, epithelial-like morphology) are encountered only in the lumen of the neural tube and, in part, integrated into the roof plate with no neural crest migration. (F) Melan A immunoreactivity confirms the melanocytic origin of the cells. (G) Schematic drawing of chick embryo 72 h after transplantation of B16-F1 melanoma cells into the optic cup. (H) Histological correlate of schematic drawing. Already in H&E staining the transplanted, invasively migrating melanoma cells are visible (arrows). (I) Single melanoma cells (identified by HMB45 immunoreactivity) form a tumor, and single melanoma cells invade the choroid of the optic cup (arrows). (J) Chick embryo 96 h after transplantation of human metastatic melanoma cells into the brain vesicle at the hindbrain (rhombencephalon). The cells form a large tumor in the dorsal neuroepithelium with (K) single HMB45 positive cells infiltrating the surrounding brain tissues. (L) MIB1 immunohistochemistry (proliferation marker not cross-reacting with chick cells) identifies melanoma cells during haematogenous spreading in blood vessels among host erythrocytes and lymphocytes, and in the surrounding neural tissue.

A major challenge is to identify single migrating melanoma cells among normal chick embryo cells. In the early chick embryo we used immunohistochemistry of melanocyte and melanoma cell specific markers HMB45 and Melan-A. Since in the early chick embryo neural crest cells have not yet differentiated, they do not express markers of the melanocyte cell lineage. This changes at E6 during emigration of neural crest cells on the lateral pathway [15]. Now the melanocyte precursors of the chick embryo also become HMB45 positive. As absolutely specific marker, in situ hybridization with the species-specific DNA sequences Alu for human and L1 for mouse cells can be performed [21]. In contrast to the mouse, there is no cross-reactivity in the chick embryo [21]. Anti-MIB-1 (Dako, Hamburg, Germany) immunohistochemistry (marking cells that are in the state of DNA synthesis) specifically stained nuclei of human melanoma cells without cross-reactivity with the chick host (Fig. 3L). Finally, we used live epi-fluorescence with murine GFP-VASP-transfected B16-F1 cells [19]. GFP-VASP labels lamellipodia and filopodia of migrating B16-F1 cells. The technique allowed the in ovo visualization of the melanoma cells emigrating from the neural crest or colonizing the optic cup [15].

### Aggregate Formation and Specific Treatment

The advantage of using aggregates instead of cell suspensions was better reproducibility of the site-specific transplantation of melanoma cells into the lumen of the neural tube, a small embryonic compartment. While melanoma cell suspensions spread out within the entire lumen of the neural tube, the aggregates remained at the site of transplantation (between the 14^th^ and the 20^th^ pairs of somites). For aggregate formation we used a roller culture procedure in gas permeable biofoil bags developed for reproducible generation of small organ cultures [22]. Cell suspensions of 10^6^ cells in 1 ml transformed into melanoma cell aggregates after rotation for 24 h [17]. During aggregation, the melanocytes were treated with BMP-2 (20 ng/ml) or nodal (30 ng/ml, both from R&D Systems, Wiesbaden, Germany).

## Results and Discussion

### Neural Crest and EMT

In the embryo, emigration of neural crest cells from the neural tube is designated as epithelial mesenchymal transition (EMT). EMT represents a complex change in cell morphology and migratory potential of embryonic cells and is induced in the embryonic neural crest by BMP and inhibited by sonic hedgehog and noggin signaling [23]. EMT comprises two consecutive steps [24]: First, the neural crest compartment is induced in the epithelium of the neural tube. This step is morphologically characterized by the disintegration of the basal lamina in the region of the lateral roof plate. Second, neural crest cells start migration from the dorsal edges of the neural tube along their medial and lateral pathways. Neural crest cells following the medial pathway form spinal ganglia and the autonomic ganglia of the sympathetic chain. The melanocyte precursors emigrate at a later stage and follow the lateral pathway. The melanoblasts then colonize the epidermis and are the source of melanocytes in the adult individual [25]. Melanomas arise from melanoblasts or melanocytes in the epidermis. Due to the neural crest lineage of melanocytic cells, the neural tube was chosen as transplantation site.

### Spontaneous Neural Crest Cell Migration of Melanoma Cells

Upon transplantation into the neural tube of the early chick embryo, melanoma cells integrated into the neural crest, performed EMT, and migrated along both pathways. EMT of melanoma cells in the neural crest environment of the chick embryo was evident when comparing the compact, round, epithelial-like morphology of the melanoma cells remaining in the lumen of the neural tube with the migrating fraction of the cells featuring a stretched, mesenchymal morphology (Figures 3A and B).

The segmental pattern of medial neural crest migration is due to inhibitory signals in the caudal and chemotactic clues in the rostral halves of the sclerotomes [25]. Transplanted melanoma cells follow the segmental medial pathway and were found in the clusters of the sympathetic chain ganglia close to the dorsal aorta [14]. In some embryos single melanoma cells were observed in the Schwann cell compartment at the motor root of the spinal nerve. The detection of single migrating melanoma cells in the chick embryo was not trivial. The entire pathway from the bulk of transplanted cells to the sympathetic ganglia is represented only in serial cross sections perpendicular to a plane tangential to the curved neural tube (Figures 3A and D). A large portion of colonized sympathetic chain becomes visible when the embryo was sectioned in the tangential plane itself [15]. In the sympathetic ganglia and in part also in the sclerotomal mesenchyme the melanoma cells undergo apoptosis as shown by tunnel and caspase-8 immunohistochemistry (Fig. 3 C; [15]).

In the chick embryo migration of neural crest cells along the lateral pathway is delayed by about 24 h as compared to the medial pathway. After EMT neural crest cells rest in the dorsal mesoderm between roof plate and surface ectoderm (“staging area” [25]). The epithelial dermomyotome portions of the somites inhibit neural crest cell migration between surface ectoderm and somites. Only after dissolution of the epithelial dermatomes, non-segmental migration along the lateral pathway starts. Melanoma cells resting amongst the chick melanocyte precursor cells in the staging area after EMT also resume neural crest cell migration along the lateral pathway. However, they are heavily decimated by apoptosis during migration so that only few can be finally detected in the lateral body wall [15]. Considering the large amount of apoptotic melanoma cells at the final spots of terminal differentiation, one can speculate that site-specific factors physiologically driving terminal differentiation in the embryo (e.g. para-aortically for neural crest cells destined to form the sympathetic chain, or below the ectoderm for melanoblasts) seem to eliminate the melanoma cells via induction of apoptosis. The apoptosis program seems to be the only way for melanoma cells to react to these site-specific embryonic micro-environmental circumstances.

### Non-transformed Primary Human Melanocytes do not Perform Neural Crest Cell Migration

To demonstrate that the neural crest migration spontaneously performed by melanoma cells upon transplantation into the neural tube was due to re-expression of embryonic traits, aggregates from primary human melanocytes were also injected into the neural tube. Histological evaluation demonstrated that the melanocyte aggregates integrated into the roof plate but did not perform neural crest migration (Figures 3D–F), indicating that the neural crest-derived melanocytes residing in the differentiated stratified epithelium of the skin have lost the capability of spontaneous neural crest migration.

### Transplantation into the Optic Cup is a Model for Invasive Migration of Melanoma Cells

As second niche for the investigation of invasion, the embryonic optic cup was chosen. Upon transplantation of B16-F1 melanoma cells into stage 19–20 HH embryos and incubation for 72 h, histological evaluation illustrated that one part of the melanoma cells had remained behind the lens at the spot of transplantation, while a second part had formed tumors in and invaded the choroid (highly vascularized, loose mesenchymal connective tissue) in the region of the prospective anterior eye chamber (Figure 3G–I). In some embryos, the cells had destroyed the lens and invaded the hyaloid vessels (not shown). Malignant growth of melanoma cells in the embryonic optic cup is also enhanced by BMP-2 and can be blocked by noggin [17].

### Transplantation into the Brain Vesicles is a Model for Brain Metastasis

As third embryonic niche for malignant growth the brain vesicles were investigated [26]. Melanoma cells were transplanted into the developing rhombencephalon (hindbrain) of the stage 12–13 HH embryo. At this stage rhombencephalic neural crest cell emigration is already completed. The location corresponds to brain liquor seeding in stage IV melanoma patients, which is associated with extremely poor outcome. In this particular niche, the transplanted melanoma cells developed a loosely formed tumor containing capillaries (not shown) after 4 days, completely destroying the dorsal roof plate and invading the surrounding mesenchymal host tissue (Figure 3J, K). Immunohistochemistry with anti-HMB45 and anti-MIB1 revealed proliferation in about 90% of the invasively growing melanoma cells (MIB1-positive, invading melanoma cells are depicted in Figure 3L). Interestingly the ventral differentiated neural plate of the rhombencephalon was excluded from invasion. Single MIB1-positive melanoma cells could be detected in blood vessels among host blood cells (Figure 3L), demonstrating that active haematogenous spreading of the transplanted melanoma cells occurred. Thus the rhombencephalic embryonic brain vesicle is an adequate model for induction and biological behavior of melanoma cells during brain metastasis.

In our previous publication [26], the focus was on different growth phases of melanoma cells. We showed that in addition to in vitro invasion (Boyden chamber and human epidermal skin reconstructs) melanoma cells from three different growth phases (radial growth phase, vertical growth phase, metastatic growth phase) retained their graded invasive qualities in vivo. For the in vivo experiment we chose the rhombencephalon as transplantation site. However, the method itself was not described in detail.

### Pre-treatment with the TGFbeta Family Members BMP-2 or Nodal Induces Invasive Migration of Human Melanocytes in the Optic Cup

**Figure 4:**
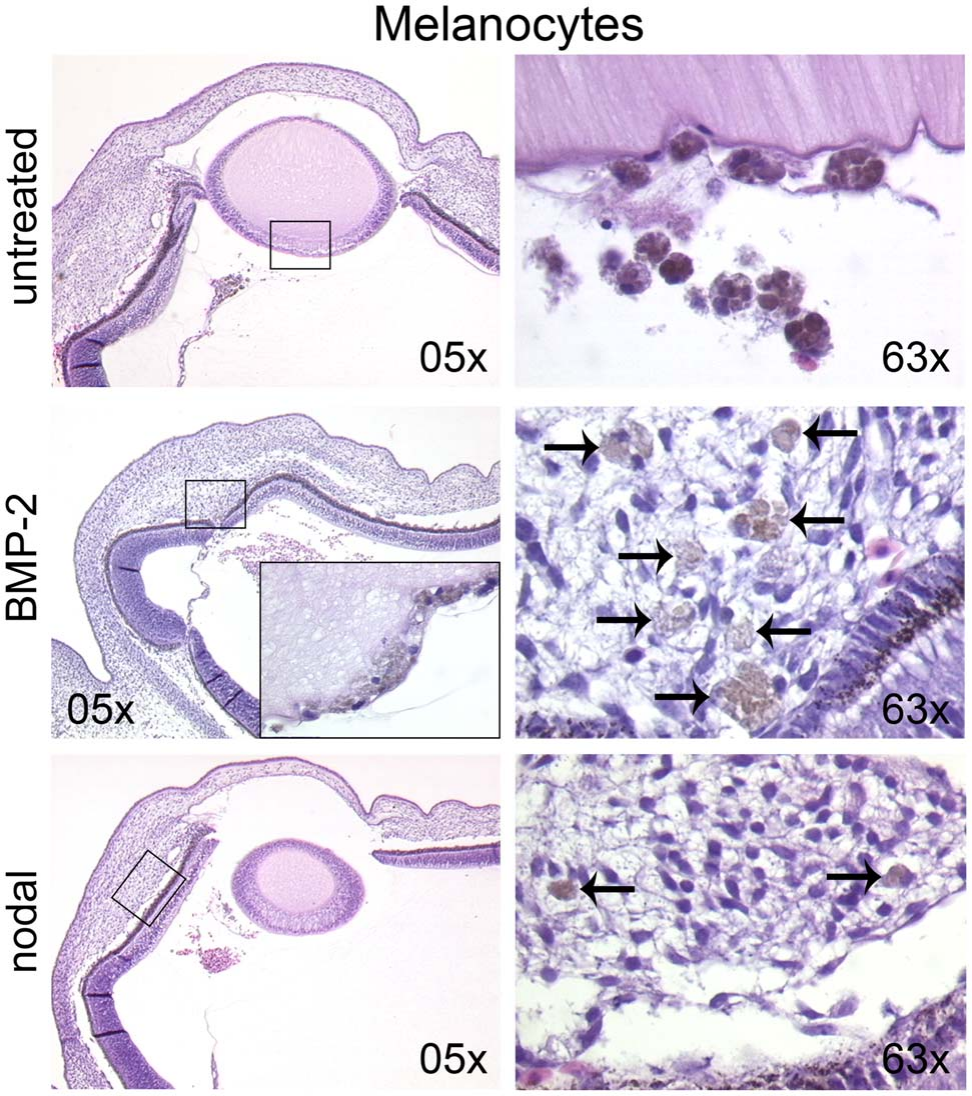
Pre-treatment with the TGFbeta family members BMP-2 or nodal induces invasive migration of human melanocytes in the optic cup. Untreated, BMP-2 or nodal pre-treated melanocytes were injected into the optic cup of the chick embryo (stage 20 HH). After 72 h of further incubation, the embryos were analyzed for tumor growth and invasion. Untreated melanocytes formed loosely aggregated tumors adjacent to the hyaloid vessels (left image in upper row), in the developing vitreous body and behind the lens (right image in upper row) without invasion. The BMP-2 and nodal groups formed tumors in similar locations. In the BMP-2 group single melanocytes invaded the lens epithelium (insert in left image in middle row), the retina, the hyaloid vessels, and the choroid (right image in middle row; arrows pointing at melanocytes). In the nodal group single melanocytes invaded the choroid (lower row, arrows in right image) and the hyaloid vessels.

We next asked whether the results gained on human melanocytes in the neural tube of the chick embryo (compare Figures 3D–F) could be re-produced in an ectopic site. We therefore injected the melanocytes into the optic cup as described above. Since in our previous reports we saw a BMP-dependence of neural crest migration of melanoma cells [16] and of invasive migration of melanoma cells in the optic cup [17], we now asked whether BMP-2 or nodal (both TGF-beta family members) could drive invasive migration in the melanocytes. As expected, untreated melanocytes formed loosely aggregated tumors behind the lens, adjacent to the hyaloid vessels and in the developing vitreous body. The human melanocytes were identified in the chick embryo by their specific pigmentation and morphology. The untreated melanocytes showed no invasion (Fig. 4, upper row). In the melanocytes pre-treated with BMP-2 or nodal we also observed the formation of loosely aggregated tumors in similar locations. In contrast to untreated melanocytes, single and groups of BMP-2 pre-treated melanocytes could be found in the lens epithelium, the retina, in the hyaloid vessels, and, most pronounced, invading the choroid (Fig. 4, middle row). In the group of nodal pre-treated melanocytes single melanocytes invading the choroid and the hyaloid vessels were observed (Fig. 4, lower row). For all three experimental groups for evaluation we grouped the melanocytes according to the compartments in which they were found: injection channel, choroid, hyaloid vessels, vitreous body, and behind the lens (compare Table 1).

### MCF7 Breast Cancer Cells Behave Differently in the Rhombencephalon than Melanoma Cells

**Figure 5:**
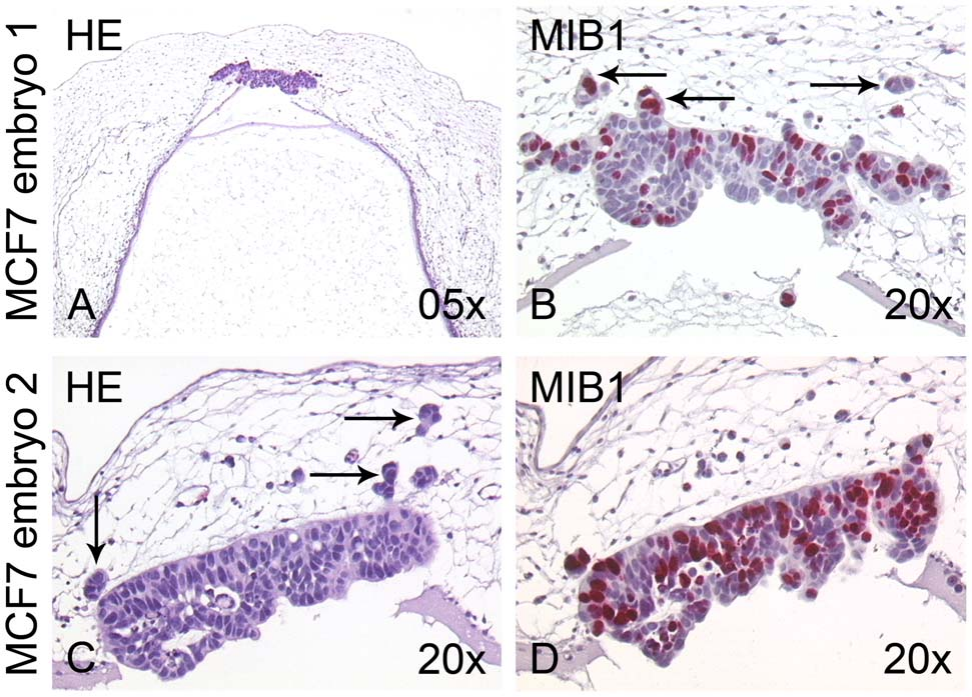
Transplantation of MCF7 breast cancer cells into the rhombencephalon of the chick embryo. (A,C) Two different examples of chick embryos 96 h after transplantation of MCF7 breast cancer cells into the rhombencephalon, H&E stainings. The cells have formed stretched, compact epithelial tumors in the roof plate and adjacent mesenchyme. (B,C) Higher magnification reveals that the MCF7 cells invade the chick host in small aggregated clusters (arrows). (B,D) MIB1 immunohistochemistry shows that 30–50% of the MCF7 cells proliferate in the chicken environment.

To analyze, whether the rhombencephalon was a transplantation site that specifically allowed melanoma cells to form invasive tumors, we injected MCF7 breast cancer cells (as cell suspension) into the same embryonic compartment (n = 7 embryos) and allowed further incubation for 96 h. Figure 5 displays two exemplary embryos transplanted with MCF7 cells. To our surprise, we encountered a different histological outcome when compared to the melanoma cells. MCF7 cells had formed compact stretched epithelial tumors in the roof plate, clearly demarcated from the host tissue (Figure 5A). Centrally the MCF7 tumors had areas with necrotic and apoptotic cells (Figure 5C). Invasion of MCF7 cells occurred in small clusters of cells (Figures 5B and C, arrows). In one case, densely aggregated MCF7 cells collectively penetrated the roof plate (not shown); invasion of the roof plate of single MCF7 cells (a phenomenon frequently observed for melanoma cells in the same context) was not found. The MCF7 cells showed less MIB1-reactivity (30–50% MIB1-positive cells; Figures 5B and D) than the melanoma cells; invading MCF7 cells were mostly MIB1-negative (as opposed to invading melanoma cells; compare Figure 3L). Interestingly, even some obviously apoptotic MCF7 cells (with nuclear fragmentation) were still MIB1-positive. Further, we could not detect any capillary sprouting into the MCF7 tumors, probably due to the compact epithelial phenotype of the tumors. This fact might account for the central necrosis visible in all of the developed tumors.

In conclusion, its feasibility, cost-effectiveness and outstanding susceptibility to manipulation with good reproducibility render the chick embryo an in vivo system to study invasion by cancer cells in an embryonic environment. It may be useful for the distinction of physiological and invasive migration of melanoma cells and melanocytes in designated embryonic niches and for the manipulation via pre-conditioning of the transplanted cells. Further, the rhombencephalic niche can also be used as model for tumor growth and malignant invasion for breast cancer cells.
